# Cultural Differences in Emotion Suppression in Belgian and Japanese Couples: A Social Functional Model

**DOI:** 10.3389/fpsyg.2020.01048

**Published:** 2020-05-27

**Authors:** Anna Schouten, Michael Boiger, Alexander Kirchner-Häusler, Yukiko Uchida, Batja Mesquita

**Affiliations:** ^1^Center for Social and Cultural Psychology, Faculty of Psychology and Educational Sciences, University of Leuven, Leuven, Belgium; ^2^Department of Psychology, Faculty of Social and Behavioural Sciences, University of Amsterdam, Amsterdam, Netherlands; ^3^School of Psychology, University of Kent, Canterbury, United Kingdom; ^4^Kokoro Research Center, Kyoto University, Kyoto, Japan

**Keywords:** culture, close relationships, emotion suppression, emotion, conflict resolution

## Abstract

Emotion suppression has been found to have negative psychological and social consequences in Western cultural contexts. Yet, in some other cultural contexts, emotion suppression is less likely to have negative consequences; relatedly, emotion suppression is also more common in those East-Asian cultural contexts. In a dyadic conflict study, we aim to (a) conceptually replicate cultural differences found in previous research with respect to the prevalence and consequences of emotion suppression, and (b) extend previous research by testing whether cultural differences are larger for some than for other types of negative emotions. We postulate that cultural differences in suppression are less pronounced for socially engaging emotions (e.g., guilt) than socially disengaging emotions (e.g., anger), because the former foster the relationship, whereas the latter emphasize individual goals. Belgian (*N* = 58) and Japanese (*N* = 80) couples engaged in a 10-min conflict interaction followed by video-mediated recall, during which participants rated their emotions and emotion suppression every 30 s. As predicted, Japanese participants reported more suppression than their Belgian counterparts, but the cultural difference was more pronounced when participants experienced more socially disengaging emotions than when they experienced more socially engaging emotions. These results suggest that the type of emotion should be considered when describing cultural differences in emotion suppression. Finally, and consistent with previous research, emotion suppression was negatively associated with interaction outcomes (i.e., conflict resolution) in Belgian couples, but not in Japanese couples.

“Unexpressed emotions will never die. They are buried alive and will come forth later in uglier ways.”

秘すれば花(世阿弥・風姿花伝“If hidden, it’s elegant”.

By Zeami, Fūshikaden

顔で笑って心で泣く(背中で泣く) “Smiling face, crying heart”

(Common Japanese Proverb)

Insights from Western folk theory and psychology alike are that emotion suppression is unhealthy. This idea, introduced by Sigmund Freud (1856–1939) (illustrated by quote above), has penetrated popular thinking, and does so even today: a simple Google search with the key term “suppression of emotions” for Belgium alone, generates 9,180,000 results. The majority of these entries express the idea that emotion suppression is unhealthy: “Suppressing negative emotions is unhealthy” and “10 dangerous things suppressing emotions can lead to” are examples. The extant psychological research on emotion regulation is consistent with this idea that emotion suppression is “unhealthy,” at least in research conducted within Western cultural contexts. Emotion suppression in these cultural contexts is found to be associated with both poor psychological outcomes and low relational well-being (Butler et al., 2003; Gross and John, 2003; Haga et al., 2009).

Emotion suppression has a better press in some other cultures, as is illustrated by the two Japanese quotes. Consistently, in many East-Asian, interdependent cultural contexts, emotion suppression is more common and less detrimental to psychological well-being and relationships than in Western cultural contexts (Butler et al., 2007; Matsumoto et al., 2008a, b; Cheung and Park, 2010; Mauss and Butler, 2010; Soto et al., 2011). These cultural differences in emotion suppression are meaningful (Mesquita and Delvaux, 2012; Mesquita et al., 2014): individuals suppress their emotions when expressing them would be negative, or at times, when suppressing is beneficial (cf. Le and Impett, 2013).

Expanding previous findings (e.g., Matsumoto et al., 2008a; Soto et al., 2011), we suggest that cultural differences not merely exist with regard to the general levels of emotion suppression, but also with respect to the specific types of emotions being suppressed. We postulate that emotions that advance the cultural relationship ideals may be expressed, even in cultures that promote emotion suppression generally (Mesquita et al., 2014). For instance, emotions that help to strengthen the bond (e.g., guilt; Baumeister et al., 1994) may be expressed; this would be the case even in interdependent cultural contexts where high levels of emotional suppression generally benefit the cultural ideal of harmony in relationships. Cultural differences in suppression are, therefore, expected to vary by emotion.

In the current study, we expect cultural differences in (a) the overall level of emotion suppression, and (b) the level of suppression for particular types of emotion; we also examine (c) the association between emotion suppression and interaction outcomes. Our predictions have been tested in the context of a cross-cultural dyadic study in the lab; we compare emotion suppression in Belgian (i.e., Western) and Japanese (i.e., East Asian) couples who engaged in a disagreement interaction.

## Suppression and the Cultural Meaning of Emotion

The central assumption guiding this research is that the extent to which romantic partners suppress their emotions in the context of disagreement depends on the cultural model of self and relationship. We compared the level of emotion suppression during couple disagreement between a Western European (i.e., Belgian) and an East Asian (i.e., Japanese) cultural context. In Western (European) cultural contexts, where independence is foregrounded, a central relational task is to discover and affirm internal attributes such as preferences, desires, or needs (Kitayama et al., 2009). In good relationships, each partner asserts and acts according to these internal attributes. Emotional expression (Rothbaum et al., 2000; Rothbaum et al., 2002; Markus and Kitayama, 2003) and self-disclosure (Chen, 1995; Kito, 2005) may be common aspects of the relationship that allow you to achieve a relationship that meets your personal preferences, desires, and needs. For instance, expressing annoyance toward your partner communicates that your personal needs have not been met. In Western cultural contexts, emotion suppression may thus be detrimental for the relationship because it hinders the assertion of independent goals.

In contrast, in East-Asian cultural contexts, in which interdependence is foregrounded, a central task for the individual is to anticipate and accommodate to the needs of others in relationships (Morling et al., 2002). In good relationships, partners act to meet others’ expectations, and to fulfil their normative role in the relationship; personal preferences, desires or needs ought to be set aside. Consequently, emotions that focus on individual preferences, desires, and needs should be concealed or hidden in order to protect the relationship. For instance, individuals are expected to suppress their annoyance over their partner’s behavior, because expressing it may draw attention to your personal needs and away from your partner’s needs, or the normative roles in the relationship. Self-expression (Kim et al., 2006) and open communication of feelings (Rothbaum et al., 2000, 2002; Markus and Kitayama, 2003) are not as valued in East Asian as they are in Western cultural contexts. Emotion suppression for the sake of relational harmony appears functional in interdependent relational contexts.

In support of cultural differences in the levels of emotion suppression during couple disagreement is research showing that emotion suppression is generally used more frequently by members from independent (e.g., Western) than interdependent (e.g., East-Asian) cultures (Gross and John, 2003; Gross et al., 2006; Safdar et al., 2009; Kim and Sasaki, 2012; Moran et al., 2013). For instance, in a study with participants from 32 countries, country-level individualism (by some authors used interchangeably with independence) predicted suppression on a bi-dimensional scale ranging from expression to suppression. High levels of individualism (as established for Western cultures) predicted more expression (and less suppression) than low levels of individualism (Matsumoto et al., 2008a). In another study, Matsumoto et al. (2008b) measured suppression in 23 countries with the suppression sub-scale of the Emotion Regulation Questionnaire (ERQ), which is unidimensional. Despite the use of a different measure of suppression than the previous study, this study yielded an association between country-level individualism and suppression as well. Again, low levels of individualism predicted higher levels of suppression. In sum, the extant research suggests that emotion suppression is more common in East-Asian than Western cultural contexts. In this study, we sought to replicate this finding for Japanese and Belgian couples during conflict interactions.

## Suppression and the Cultural Meaning of Disengaging and Engaging Emotions

The current study goes beyond prior research, to propose that cultural differences in emotion suppression also depend on the type of emotion. We distinguish between two types of emotions that form the extremes of the dimension of social engagement: socially disengaging and socially engaging emotions (Kitayama et al., 2000). Social engagement defines the emotion domain across different cultures. On the disengaging end, emotions such as anger (negative) and pride (positive) underline the self as separated from others, whereas on the engaging end, emotions such as guilt (negative) and friendly feelings (positive) help to connect the self with others in the relationship.

Unwittingly, cross-cultural studies on emotion suppression have largely focused on the suppression of disengaging emotions, at the expense of engaging emotions. For example, the study by Matsumoto et al. (2008a) included negative disengaging emotions like anger, contempt and disgust, in addition to some positive emotions and sadness; no negative engaging emotions (e.g., shame, worry, and guilt) were included. The study revealed that participants high on individualism expressed their emotions more (and suppressed them less) than participants low on individualism. One exception was sadness: participants high on individualism suppressed sadness more than participants low on individualism, which is neither engaging nor disengaging. One question is, therefore, whether negative engaging emotions would be equally suppressed in cultures low on individualism? It is conceivable that emotion suppression is marshaled to mitigate negative disengaging but not negative engaging emotions, as only the former are considered harmful to interdependent relationships. Especially in the context of a disagreement interaction, we expect negative disengaging emotions to be acceptable in Belgium where they help partners to assert and protect their individual goals. In contrast, in Japan where preferences, desires and needs should be put aside during disagreement, negative disengaging emotions work crossways.

In the current study, we broaden the research on cultural differences in emotion suppression to include negative engaging, in addition to negative disengaging emotions. We predict that, especially during a disagreement interaction, cultural differences in emotion suppression are larger for negative disengaging than engaging emotions. We predict that partners in Japan use more emotion suppression generally, but do not suppress their negative engaging emotions more than their Belgian counterparts. Conversely, cultural differences in emotion suppression should be larger for disengaging emotions, as these emotions have a contrasting function for the relationship across cultures.

## Cultural Differences in the Consequences of Emotion Suppression

Consistent with the idea that emotion suppression is detrimental in Western cultural contexts, research within these cultures has found an association between emotion suppression and poor wellbeing – depression, low self-esteem, low life satisfaction (Gross and John, 2003; Haga et al., 2009), and the experience of fewer positive and more negative emotions – as well as negative relationship outcomes (Butler et al., 2003; Gross and John, 2003). In one study, dyads of European American female undergraduates who discussed their thoughts and feelings after having watched an emotion-eliciting movie, reported less “rapport” and lower relationship satisfaction when one partner was instructed to suppress her thoughts and feelings. Rapport and relationship satisfaction were reported by the partner who had not received the suppression instruction. This suggests that emotion suppression has both negative relational and psychological consequences in Western cultural contexts.

Emotion suppression may be less costly in East-Asian cultural contexts. One cross-cultural study with Asian and European American participants found that the relationship between anger suppression and depression varied by cultural self-construal. Whereas anger suppression was associated with depression in participants with independent self-construals, the association was much lower in participants with interdependent self-construals (Cheung and Park, 2010). Generalizing this finding to the cultural context might suggest that the consequences of anger suppression are less detrimental in cultures with an interdependent (as opposed to independent) model of self. Consistently, in a cross-cultural questionnaire study, European American participants who reported suppressing their emotions tended to be more depressed and less satisfied with life than those who did not; in contrast, no differences in depression and life satisfaction were found between East-Asian participants who did and did not report suppressing their emotions (Soto et al., 2011). One reason for cultural differences in the consequences of suppression may be its differential effect on the experience of the emotion. Suppression reduces the experience of unwanted emotions (i.e., anger) in East-Asian, but not in Western cultural contexts (Mauss and Butler, 2010; Allen et al., 2014). More specific to close relationships, the negative relational consequences of emotion suppression also appear to vary across cultural contexts. In a dyadic suppression study similar to the one described above, suppression was less detrimental to the relationship in Asian American than European American dyads (Butler et al., 2007). The partners of European American suppressors perceived the suppressor as more hostile and withdrawn, and responded to the suppressor with more hostile behavior than the partners of Asian American suppressors.

Results are mixed, though. In the dyadic study, suppression had some unfavorable effects, even in Asian American dyads: Asian and European American partners alike indicated that they were unwilling to pursue a friendship with the suppressor, and displayed fewer affiliative behaviors toward them. Furthermore, in a cross-cultural survey study using the ERQ, emotion suppression was associated with lower relationship satisfaction in American as well as Chinese individuals (English and John, 2013); the suppression items of the ERQ do not specify emotions. Against the background of the mixed findings on cultural differences in the relational consequences of emotion suppression, we will study these consequences in the context of couple disagreement.

## Present Research

The present study focuses on emotion suppression during couple disagreements. It compares couples from two different cultural contexts – Belgium and Japan – that are on opposite ends of different scales of independence vs. interdependence. For example, Schwartz (2006) found that Belgian samples were high on autonomy (e.g., being independent), whereas Japanese samples were high on embeddedness (e.g., interdependence in relationships). Furthermore, Belgian samples were high on egalitarianism, another facet of independence, whereas Japanese samples were high on hierarchy (e.g., pertaining to ascribed roles), considered a facet of interdependence. The research focuses on cultural differences in suppression, contrasting Belgian and Japanese partners. Building on as well as expanding previous research on cultural differences in emotion suppression, we tested the following three hypotheses:

1. Japanese partners endorse more emotion suppression than Belgian partners during disagreement.
2. Compared to Belgian partners, Japanese partners of couples report more emotion suppression when negative disengaging emotions take precedence over negative engaging emotions no such differences in emotion suppression are expected when negative engaging emotions take precedence over negative disengaging emotions.
3. Emotion suppression is negatively associated with interaction outcomes in Belgian partners, but not in Japanese partners.

We tested these hypotheses using data from a lab study on couple conflict^1^. We focused on couple conflict as the context to study emotion suppression, because within conflicts, emotions and emotion suppression are on display. Moreover, this context allowed us to build on a large literature describing emotions during relationship-conflict (e.g., Levenson et al., 2014), and to measure emotion suppression in response to actual emotions triggered in a standardized interaction. We focused on negative disengaging and engaging emotions, the types of emotions central to our hypotheses.

## Methods

### Participants

58 Belgian participants from in and around the city of Leuven and 80 Japanese participants from Kyoto/Osaka area were recruited for the study in turn for a monetary price. The present study is part of a larger study on emotions during couple conflict. For the larger study, sample sizes were collected that far exceeded those of previous studies also using an interaction paradigm (Tsai et al., 2006; Lee et al., 2013; Hiew et al., 2015). All participants that enrolled in the larger study were included in the analyses for this study. Participants were (a) between 30 and 50 years old and (b) native Dutch/Japanese speakers. Partners were selected to have been in a heterosexual relationship for at least 2 years, and cohabiting. Due to technical difficulties, one Belgian couple and four Japanese participants had to be excluded, thereby leaving the data of 57 Belgian and 76 Japanese participants for analyses.

In the final sample, Belgian participants were significantly younger than Japanese participants [*M*_*BE*_ = 41.22, *SD*_*BE*_ = 5.15; *M*_*JP*_ = 42.98, *SD*_*JP*_ = 4.33, *t*(221.20) = 3.00, *p* < 0.01]. No cultural differences were found in terms of relationship duration: on average, both Japanese and Belgian participants had been in a romantic relationship for approximately 15 years [*M*_*BE*_ = 15.55, *SD*_*BE*_ = 8.20; *M*_*JP*_ = 14.98, *SD*_*JP*_ = 7.20; *t*(136) = 0.44, *p* = 0.66]. The majority of participants in both countries were married but, reflective of the difference in cultural practice, a higher proportion of Japanese (97.5%) than Belgian participants (72.4%) were married, χ^2^(1) = 18.66, *p* < 0.001.

### Procedure

We focus the discussion of the Methods on the relevant parts for this research (the full package of material for the larger study can be consulted in the Supplementary Tables S1, S2). In the main part of the study, couples participated in a dyadic interaction task that was originally developed by Levenson and Gottman (1983) for research on marital processes. For the current study, the procedure of the dyadic interaction task was slightly adjusted to ensure applicability in Japanese and Belgian contexts. In the original procedure developed by Levenson and Gottman (1983), a research assistant (RA) picked the conflict topic that elicited the largest emotional response during an exploratory conversation, in which the couple was asked to briefly touch on the various conflict areas that they had independently indicated as important in a prelab questionnaire. We tried this procedure in a pilot, but it soon became clear that Japanese participants experienced it a breach of confidence that the RA brought up areas of conflict that they had confidentially, and unbeknownst to the other, listed in a questionnaire. We changed the procedure to accommodate these concerns, and instead compiled a list with all conflict areas that at least one of the partners had rated as higher than zero on the scale. No mention was made of the origin of these topics, and the couple was simply asked to choose which area of disagreement they were going to discuss during the lab session. This procedure ensured that confidential information was not revealed to the partner and that the couple’s choice to discuss a topic (or not) was respected. The entire conflict interaction study consisted of three steps: the pre-laboratory phase, the dyadic interaction task at the lab, and the video-mediated recall, also at the lab.

#### Pre-laboratory Phase

Each partner completed an online questionnaire package at home at least three days prior to the lab visit. The package included measures of relationship satisfaction and areas of couple conflict. Partners were asked to fill out the questionnaire independently.

#### Dyadic Interaction Task

Partners engaged in three interactions in the lab that were each video-recorded: (a) a neutral interaction (5 min) during which they talked about a random and current event in their lives; (b) a conflict interaction (10 min); and a positive interaction (10-min) with the purpose of resolving any remaining tension between the partners. The conflict interaction is the focus of the current study. First, couples selected an area of disagreement from the compiled list of disagreement areas. Before the start of the interaction, the RA gave the couple several instructions: couples were asked (1) to behave naturally, as if they were at home (2) to recall the last time they had had a disagreement about the selected topic and (3) to start their conversation by each stating their point of view. In addition, couples were encouraged to try and solve the problem. A chime signaled both the beginning and end of the conversation.

#### Video Mediated Recall

After the dyadic interaction tasks, partners were led into separate rooms to participate in a video mediated recall (VMR). The VMR allowed for a measure of emotion suppression in response to momentary and real emotions during ongoing social interactions. Each partner viewed the recording of their conflict interaction two times, the first of which will be reported here. During the first viewing, the video recording was stopped every 30 s, and participants indicated to what extent they had (a) experienced each of 12 discrete emotions and (b) suppressed their emotions. Before the start of the actual VMR, participants completed a short training session to familiarize them with the procedure. The VMR software malfunctioned for one Belgian couple (3020) and four Japanese participants (2011, 2012, 2051, and 2053), leaving us with 57 Belgian and 76 Japanese participants for analyses. Immediately after the VMR, participants were asked to fill out a questionnaire about the conflict interaction, which included an item on conflict resolution.

### Measures

#### Negative Disengaging and Engaging Emotions

Immediately after the dyadic interaction task, participants were asked to rate 12 emotions while reviewing their conflict interaction in the VMR. These emotions were selected based on a preliminary study that ensured their cross-cultural relevance. In this study, Japanese and Belgian participants in a relationship rated a recent conflict interaction on 48 emotions (a) taken from previous research on emotional experience during conflict (Bell and Song, 2005; Coan and Gottman, 2007; Sanford, 2007) and between romantic partners (Gonzaga et al., 2007) (b) representing major dimensions of emotional experience (Watson et al., 1988; Fontaine et al., 2013) and (c) representing emotional dimensions that differ significantly in prevalence across cultures (Mesquita, 1993; Scherer, 1997; Kitayama et al., 2000; De Leersnyder et al., 2011; Boiger et al., 2013a, b). Using Clusterwise simultaneous component analysis (De Roover et al., 2012), an analytical method that establishes if a component solution (similar to a factor structure) holds across all blocks (i.e., all cultures), we found a common solution across cultures. From this common solution, we selected 12 emotions that scored the highest and/or were the most theoretically relevant: eight negative emotions (resigned, hurt, annoyed, aloof, afraid of hurting my partner, guilty, worried, and embarrassed) and four positive emotions (amae/like my partner would indulge any of my requests, empathy for my partner, strong, and calm).

To establish whether positive and negative disengaging and engaging emotions loaded on different factors in the current study, we conducted principle component analysis (PCA) with the raw data derived from the VMR; we used the emotion ratings of each partner per time point^2^. We excluded amae because it formed its own factor. PCA on the pooled data (i.e., taking into account both cultures) yielded three components: negative disengaging emotions, negative engaging emotions, and positive emotions. Our hypotheses pertained to the negative emotions only. For the purposes of this study, we assigned all eight negative emotions to one of the two categories of negative emotions: negative disengaging and negative engaging emotions. Assignment to a category was based on factor loadings; cross-loadings were found for hurt and embarrassed, which were assigned to either category based on conceptual considerations (Table 1 shows the two categories:

**TABLE 1:** Negative disengaging and engaging emotions.

	Factor loadings
**Negative disengaging emotions**	
Resigned	0.566
Hurt	0.698
Annoyed	0.839
Aloof	0.662
**Negative engaging emotions**	
Afraid of hurting my partner	0.757
Guilt	0.795
Worried	0.629
Embarrassed	0.404

α_JP disengaging emotions_ = 0.82, α_JP engaging emotions_ = 0.79, α_BE disengaging emotions_ = 0.61, α_BE engaging emotions_ = 0.73). We calculated the average emotion rating of negative disengaging and engaging emotions for each partner per time point, and next, we averaged the ratings across time points, yielding one rating for disengaging and one rating for engaging emotions per partner.

Finally, we calculated each individual’s tendency to feel negative disengaging vs. engaging emotions. This was done by subtracting the score for negative engaging emotions from the score for negative disengaging emotions for each participant. We refer to the resulting score as *disengaging* vs. *engaging emotions*. High scores on disengaging vs. engaging emotions indicate higher levels of negative disengaging than negative engaging emotions, and low scores indicate higher levels of negative engaging than negative disengaging emotions. The disengaging vs. engaging emotions score was calculated for several reasons. First, people tend to experience engaging and disengaging emotions simultaneously; the two types of emotions may be positively correlated. Indeed, we found a positive correlation between disengaging and engaging emotions in both Japan (*r* = 0.76) and Belgium (*r* = 0.54). Because multicollinearity hinders unique estimation of regression coefficients – it is impossible to estimate the effect of disengaging emotions on suppression controlling for engaging emotions and vice versa – we used the disengaging vs. engaging emotions score for further analyses. Second, the disengaging vs. engaging emotions score allows for insight into emotion suppression when one or the other emotion prevails, and as such, allows us to examine if cultural differences in emotion suppression are larger when disengaging emotions take precedence over engaging emotions.

#### Emotion Suppression

Emotion suppression was measured by a single item that asked about the extent to which participants wanted to hide their emotions; the scale ranged from 0 (*not at all*) to 6 (*very much*). Participants rated emotion suppression 20 times, namely every time the video recording stopped (every 30 s). The average emotion suppression across different time points constituted a participant’s suppression score.

#### Interaction Outcomes: Conflict Resolution

We considered conflict resolution as a relevant interaction outcome in the context of disagreement. Conflict resolution was measured immediately after the VMR by a single item that probed the extent to which partners had come to “a solution or compromise during the conversation”; the scale ranged from 0 (*not at all*) to 6 (*very much*).

### Control Measures

#### Conflict Areas

To select a meaningful topic for the conflict interaction, participants completed an adapted version of the Couple’s Problem Inventory (CPI; Gottman et al., 1977) during the pre-laboratory assessment. The total number of conflict areas in the adjusted CPI was 22 (e.g., children, sex, and household-related issues); the original items were supplemented with items from the Dyadic Adjustment Scale (Spanier, 1976) and with items tailored to the Japanese context. Participants indicated the degree of disagreement for each conflict area on a scale from 0 (*no disagreement at all*) to 100 (*high disagreement*). They also had an option of adding any additional area of conflict that was not on the list. Areas of conflict rated higher than zero by either partner were incorporated in a list from which the couple were to choose a topic for the lab interaction.

#### Relationship Satisfaction

To assess relationship satisfaction in both cultures, we administered the 16-item Couple Satisfactory Index (CSI; Funk and Rogge, 2007) and an additional item on perceived emotional support (Uchida et al., 2008) that was suggested by the Japanese team. As in the original CSI, the first item (“*Please indicate how you would judge the degree of happiness in your relationship*”) was rated on a seven-point scale. Also consistent with the original CSI, all other items were rated on six-point scales ranging from 0 to 5 (0 was the lowest and 5 the highest score; scale points were defined in slightly different ways for different items). Sample items were “*I really feel like part of a team with my partner*” and “*I feel miserable about my relationship*” (reverse-coded). The item suggested by the Japanese team was “My relationship is *respectful*.” Cronbach’s alpha’s for our 17-item relationship satisfaction scale were 0.96 in Belgium and 0.95 in Japan.

## Results

### Statistical Analysis

To test our hypotheses, we used multilevel models (MLM) for dyadic data analyses (Kenny et al., 2006) with SPSS. Couples were situated at level 2 and participants were situated a level 1; partners were nested in the couple and individual scores were treated as repeated measures. Culture was treated as a level 2 variable for each model and transformed to a dummy variable. To test whether the level of emotion suppression differed across cultures (hypothesis 1), we solely entered culture as a predictor variable of emotion suppression. To test the relationship between disengaging vs. engaging emotions and emotion suppression across cultures (hypothesis 2), disengaging vs. engaging emotions, culture and the interaction between disengaging vs. engaging emotions and culture were entered into the model as predictors of emotion suppression. The variable “disengaging vs. engaging emotions” was treated as a level 1 independent variable and centered around the culture mean. To test the relationship between emotion suppression and conflict resolution across cultures (hypothesis 3), emotion suppression, culture and the interaction between emotion suppression and culture were entered into the model as predictors of conflict resolution. Emotion suppression was treated as a level 1 independent variable and centered around the culture mean. For each model, we assumed a random intercept; in the model for hypothesis 2 emotion suppression, and in the model for hypothesis 3 conflict resolution could vary between couples. Non-independence between partners was modeled through this random intercept and estimated as a correlation. We did not assume a random slope because there were not enough data points per dyad. The variance between couples was 17% and the variance within couples was 83%.

Hypothesis 1:

Level 1: Emotion Suppression_*ij*_ = β_0__*j*_

Hypothesis 2:

Level 1: Emotion Suppression_*ij*_ = β_0__*j*_ + β_1__*j*_Disengaging vs. Engaging Emotion_*ij*_ + ε_*ij*_

Hypothesis 3:

Level 1: Conflict Resolution_*ij*_ = β_0__*j*_ + β_1__*j*_Emotion Suppression_*ij*_ + ε_*ij*_

Level 2: β_0__*j*_ = γ_00_ + γ_01_Culture_*j*_ + ε_0__*j*_

Level 2: β_1__*j*_ = γ_10_ + γ_11_Culture_*j*_

### Manipulation Checks

To ensure that the conflict interactions resulted in comparable conflict, we conducted several manipulation checks. First, we checked whether the level of disagreement for the selected conflict topic was similar for Japanese and Belgian couples. Although different areas of disagreement were discussed (see online Supplementary Table S3 for an overview) [χ^2^(17) = 29.50, *p* = 0.010, Fisher’s exact test], the level of disagreement did not differ between the two cultures [*M*_*BE*_ = 31.98, *SD*_*BE*_ = 20.43; *M*_*JP*_ = 34.40, *SD*_*JP*_ = 20.21, *t*(131) = 0.497, *p* = 0.50]; both Japanese and Belgian couples chose to discuss topics on which they moderately disagreed. Next, we checked if the disagreement paradigm tapped into processes that were relevant to the relationship. In both cultures, we found that couples who discussed more intense disagreements, also reported lower levels of relationship satisfaction (*ß_*BE*_* = −0.54, *t* = −4.80, *p* < 0.001; *ß_*JP*_* = −0.51, *t* = −5.13, *p* < 0.001).

### Do Japanese Suppress Their Emotions More Than Belgians? (Hypothesis 1)

Japanese partners suppressed their emotions marginally more than Belgian partners [*M*_*JP*_ = 0.79, *SD*_*JP*_ = 1.10, *range* = 4.15; *M*_*BE*_ = 0.52, *SD*_*BE*_ = 0.68, *range* = 5.50; *B* = 0.27, *t*(133) = 1.98, *p* = 0.050]. Our first hypothesis – that Japanese partners would suppress more than Belgian partners – was not fully borne out, therefore.

### Do Cultural Differences in Suppression Vary by Type of Emotion? (Hypothesis 2)

We hypothesized that Japanese partners would suppress their disengaging emotions, but not their engaging emotions, more than Belgian partners. The results offer support for this hypothesis: culture moderated the relationship between disengaging vs. engaging emotions and emotion suppression [*B* = 0.33, *t*(265.72) = 1.99, *p* = 0.047, 95% CI (−0.005, 0.66)]^3^, and this relationship was marginally significant for Japanese partners [*B* = 0.20, *t*(257.22) = 1.685, *p* = 0.093, 95% CI (−0.033, 0.42)], but not significant for Belgian partners [*B* = −0.13, *t*(262.56) = −1.15, *p* = 0.25, 95% CI (−0.37, 0.10)]^4^ ^,5^. Notably, in the model used for hypothesis 2, culture significantly predicted emotion suppression (this was not the case in the model used for hypothesis 1); Japanese partners suppressed more than Belgian partners when controlling for disengaging vs. engaging emotions [*B* = −0.27, *t*(128.10) = −2.80, *p* = 0.047, 95% CI (−0.53, −0.003)].

**FIGURE 1:**
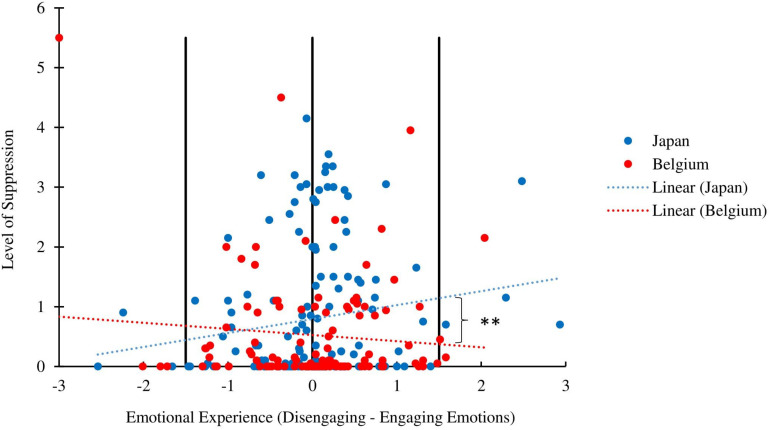
Graph depicting the relationship between the reported intensity of the emotional experience (equal to disengaging emotions minus engaging emotions) and the level of emotion suppression for Japanese (blue) and Belgian (red) partners. Reference lines are located at the mean, and at 2 standard deviations above and below the mean. ^∗∗^*p* < 0.01.

To determine whether Japanese partners suppressed more than Belgian partners at high levels of disengaging vs. engaging emotions (i.e., when disengaging emotions take precedence over engaging emotions), but not at low levels of disengaging vs. engaging emotions (i.e., when engaging emotions take precedence over disengaging emotions), we probed the interaction. More specifically, we conducted a “pick a point” analysis to establish the effect of culture on emotion suppression for particular values of disengaging vs. engaging emotions. For this analysis, we centered disengaging vs. engaging emotions at two standard deviations below and two standard deviations above the mean (Hayes, 2013). As Figure 1 illustrates, Japanese partners suppressed their emotions significantly more than Belgian partners [*B* = 0.77, *t*(249.49) = 2.710, *p* = 0.007, 95% CI (0.20, 1.32)] at high levels of disengaging emotions vs. engaging emotions (+2 SD), but not at low levels of disengaging vs. engaging emotions (−2 SD) [*B* = −0.23, *t*(249.53) = −0.81, *p* = 0.42, 95% CI (−0.79, 0.33)]^6^.

To check if gender was interchangeable, we added *gender* as well as the interaction between *gender* and *disengaging* vs. *engaging emotions* to the model as predictors. We found that gender did not moderate cultural differences; the three-way interaction (culture × disengaging vs. engaging emotion × gender) was not significant. However, for men, but not women, culture significantly moderated the relationship between disengaging vs. engaging emotions and emotion suppression: Japanese men suppressed more than Belgian men when disengaging emotions took precedence over engaging emotions [*B* = 0.62, *t*(133.30) = 2.21, *p* = 0.029, 95% CI (0.064, 1.17)], but no differences in emotion suppression were found between Japanese and Belgian women who experienced more disengaging than engaging emotions. Notably, the results for women were in the same direction as those of men, such that the relationship between disengaging vs. engaging emotions and emotion suppression was positive and marginally significant in Japanese women [*B* = 0.20, *t*(133.75) = 1.73, *p* = 0.086, 95% CI (−0.029, 0.44)], but positive and *non-significant* in Belgian women. We may thus conclude that the effect of culture on the relationship between disengaging vs. engaging emotions and emotion suppression is primarily carried by men.

### Emotional Suppression and Interaction Outcomes (Conflict Resolution) (Hypothesis 3)

**FIGURE 2:**
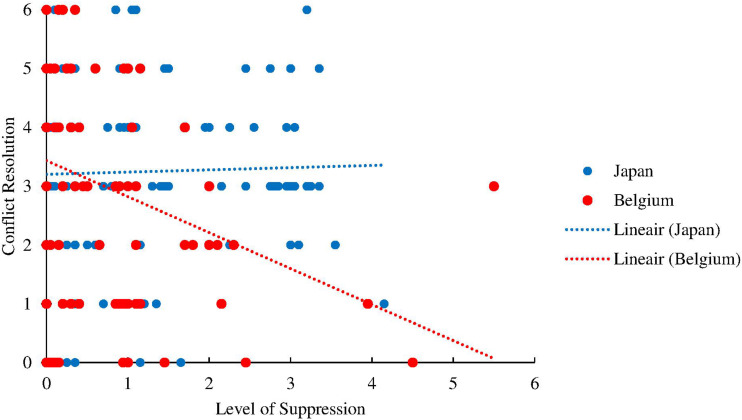
Graph depicting the relationship between the reported level of emotion suppression and conflict resolution in Japanese (blue) and Belgian (red) partners.

We expected that suppression would be associated with low conflict resolution in Belgian partners but not in Japanese partners. Consistent with our hypothesis, the results yielded a significant interaction between culture and suppression [*B* = −0.54, *t*(260) = 2.458, *p* = 0.015, 95% CI (0.11, 0.97)], suggesting that high levels of suppression are associated with low conflict resolution in Belgian partners [*B* = −0.53, *t*(261.59) = −2.968, *p* = 0.003, 95% CI (−0.88, −0.18)], but not in Japanese partners [*B* = 0.01, *t*(218.99) = 0.085, *p* = 0.933, 95% CI (−0.24, 0.26)] (Figure 2)^7^ ^,8^.

## Discussion

Central to this study was the idea that cultural differences in emotion suppression depend on the type of emotion that is experienced. More specifically, we expected larger cultural differences in emotion suppression for disengaging than for engaging emotions. In this study, we examined emotion suppression as reported during couple interactions of disagreement. In support of our hypothesis, we found that Japanese and Belgian partners who experienced more disengaging emotions (resigned, annoyed, aloof, and hurt) than engaging emotions (guilt, embarrassment, afraid to hurt partner, and feeling worried) differed with regard to the level of emotion suppression reported during a conflict interaction – Japanese partners reported more suppression than Belgian partners when disengaging emotions prevailed. However, Japanese and Belgian partners who experienced more engaging than disengaging emotions did not differ in the level of reported emotion suppression. Cultural differences in emotion suppression were in the same direction for men and for women, but the effect was stronger for men. In addition, we found that positive emotions were suppressed to different extents across cultures: Japanese partners suppressed their positive emotions more than Belgian partners did. Cultural differences were thus particularly large for disengaging (rather than engaging) emotions, as well as for positive emotions.

Our findings go beyond the existing research on cultural differences in emotion suppression by considering the type of emotions. Whereas existing studies sought to examine cultural differences in emotion suppression in general (Matsumoto et al., 2008a, b; Moran et al., 2013), we predicted that emotion suppression would differ depending on the functionality of the emotion type. Based on previous cross-cultural work on emotions, we suggested that disengaging emotions may be conducive to the goal of autonomy in Western cultural contexts (i.e., United States and Belgium), but may interfere with relational harmony in East-Asian cultural contexts (i.e., Japan) (Kitayama et al., 2000; Mesquita, 2001; Boiger et al., 2013a, b; De Leersnyder et al., 2017; Mesquita et al., 2017). Cultural differences in emotion suppression may thus be significant for disengaging emotions. In contrast, we predicted that the levels of suppression would vary less across cultures for engaging emotions, because these emotions are conducive to relational harmony in Japan. Our findings are consistent with the idea that cultural differences in emotion suppression occur when disengaging emotions dominate, but not when engaging emotions do.

In addition, this study aimed to conceptually replicate cultural differences in the prevalence of emotion suppression, as they had been found in previous comparisons between participants from interdependent and independent contexts (Gross and John, 2003; Gross et al., 2006; Matsumoto et al., 2008a, b; Kim and Sasaki, 2012). Although cultural differences in the levels of self-reported suppression were in the predicted direction – Japanese participants did report higher levels of suppression than their Belgian counterparts – they did not reach significance. An explanation for the failure to fully replicate previous findings may be precisely that the current study included engaging, in addition to disengaging emotions. In fact, we found cultural differences in emotion suppression when disengaging emotions took precedence over engaging emotions, but *not* when engaging emotions took precedence over disengaging emotions. Previous studies focused on disengaging emotions only. That the failure to replicate significant differences in emotion suppression is due to inclusion of engaging emotions is supported by our results. When we controlled for the effect of emotions, the cultural differences in emotion suppression did turn significant: Japanese partners reported significantly higher levels of emotion suppression than Belgian partners. The inclusion of a wider range of emotions in the current study may thus account for lower differences in the overall levels of emotion suppression between Japanese and Belgian partners.

A final aim of the current study was to conceptually replicate cultural differences in the interactional correlates of emotion suppression. In line with earlier research showing that emotion suppression leads to fewer negative relationship outcomes in East-Asian than in Western cultural contexts (Butler et al., 2007), we found that emotion suppression did not stand in the way of solving conflict in Japanese partners, while in Belgian partners it did. Our finding challenges the prominent and largely “Western” point of view that emotion suppression is unhealthy. As the quotes in the beginning of this article suggest, emotion suppression may actually be relatively more adaptive in East-Asian cultural contexts.

### Limitations

The current study has at least three limitations. First, and due to the low co-occurrence of suppression and disengaging/engaging emotions (co-occurrence only in 35% of the measurement points in the Japanese sample, and 27% in the Belgian sample), our multilevel analyses were based on time-aggregated data per person. Modeling the non-aggregated data yielded no interpretable results. One reason for this may have been that measurements were not continuous, and that partners only reported their emotions every 30 s. It is possible that the peaks of either the emotions or suppression itself did not neatly coincide with each other. Future research using continuous measurement may yield a more precise picture.

A second limitation of the current study is that emotion suppression was measured with a single item. A multiple-item scale would have increased reliability.

A third limitation was that participants reported the extent to which they had hidden their emotions in general, instead of how much they had suppressed either a specific emotion or the highest intensity emotions of the past 30 s. Future research should include more precise measurements of the suppressed emotions, which may also compensate for some of the co-occurrence problems mentioned above. However, the video-mediated recall constrains the number of items (pilot testing revealed that 12 emotions for 20 times was the upper limit) which complicates adding extra items to the specific design. To gain further insight in the process of emotion suppression during interactions, future research should consider including behavioral measures of suppression.

### Conclusion

This study suggests that cultural differences in emotion suppression vary by emotion type. We compared emotion suppression of Japanese and Belgian couples during conflict interactions. Cultural differences in emotion suppression were larger when disengaging emotions rather than engaging emotions were foregrounded. It is suggested that the suppression of emotions is generally valued more in Japanese than in Belgian relationships, but that expression of engaging emotions may be conducive to interdependence in Japanese relationships; engaging emotions may therefore not be suppressed as much. The finding from previous research that emotion suppression is more common in East-Asian (Japanese partners) than Western cultural contexts (Belgian partners) did not reach conventional significant levels, possibly because this study – in contrast to previous ones – included engaging emotions, in addition to disengaging emotions. Finally, emotion suppression was associated with poor interaction outcomes (i.e., conflict resolution) in Belgian, but not in Japanese couples.

## Data Availability Statement

The datasets generated for this study are available on request to the corresponding author.

## Ethics Statement

The studies involving human participants were reviewed and approved by Social and Societal Ethics Commitee, KU Leuven. The patients/participants provided their written informed consent to participate in this study.

## Author Contributions

AS has analyzed the emotion suppression data of the couple conflict study and has written the manuscript. MB and AK-H set up and collected data for the larger project on couple conflict which includes this smaller study on emotion suppression. AK-H also helped with the analyses of the emotion suppression data. YU, head of the Cultural Psychology Lab at the Kokoro Research Center, has been collaborating with us for many years. She contributed her expertise on culture, emotions and social relationships, and oversaw data collection in Japan. BM, head of the Center of Social and Cultural Psychology, contributed her expertise on culture, emotions and social relationships. She also oversaw data collection in both Belgium and Japan and critically revised the manuscript.

## Conflict of Interest

The authors declare that the research was conducted in the absence of any commercial or financial relationships that could be construed as a potential conflict of interest.

## Footnotes

1 Data from the same study were used in other research reports (Boiger et al., 2019; Kirchner, 2019). The current study is the only one to make use of the emotional suppression data.

2 We also conducted PCAs with processed rather than raw data (couple centered and within-culture standardized data, as well as person-centered data) to account for dependency across repeated measures, but these solutions were hard to interpret. Note that PCA at the couple level (using the couple’s average emotion ratings) yielded the same solution as the PCA with the raw individual-level data.

3 Degrees of freedom are decimals because of the Satterthwaite Approximation by SPSS.

4 Because positive emotions could not be assigned to either category of disengaging or engaging, emotions, we did not focus on positive emotions in the paper. However, positive emotions were suppressed differently across cultures: Japanese participants suppressed their positive emotions significantly more than Belgian participants [significant interaction effect of culture × positive emotions; *B* = 0.28, *t*(265.11) = 2.273, *p* = 0.024, 95% CI (0.038, 0.53)].

5 We examined cultural differences for each type of engaging emotion, and found that only embarrassment was suppressed less by Japanese than by Belgian partners.

6 We found the same result when probing the interaction at 1 SD above and below the mean of disengaging vs. engaging emotions.

7 We also checked if culture moderated the association between emotion suppression and relationship satisfaction, however, the interaction was not significant [*B* = −2.44, *t*(233.45) = −1.46, *p* = 0.15, 95% CI (−5.75, 0.86)].

8 Conflict resolution correlates positively with relationship satisfaction in Belgian partners (*r* = 0.27) but it does not correlate significantly with relationship satisfaction in Japanese partners.
