# Supplementary material for: Cultural Differences in Emotion Suppression in Belgian and Japanese Couples: A Social Functional Model
Source: Front Psychol. 2020 May 27;11:1048. doi: 10.3389/fpsyg.2020.01048 (PMC7326130; doi:10.3389/fpsyg.2020.01048)
Supplement: Supplementary file 1 [file Table_1.docx]

**Supplementary Online Material**

Table S1. Pre-Laboratory Questionnaires

| **Scale** | **Source** |
| --- | --- |
| Sympathy Scale | Uchida & Kitayama, 2001 |
| PANAS | Watson, Clark, & Tellegen, 1988 |
| Autonomy, Relatedness, Conformity Scale |  |
| Autonomy was measured with the 7-item autonomy subscale of the Basic Needs Satisfaction in General Scale (BNSG-S) | Johnston & Finney, 2010 |
| Relatedness was measured with 9 relatedness items of the Self Construal Scale (SCS) and 3 items of the Relational Interdependent Self-Construal Scale (RISC) | SCS: Kagitcibasi, 2007  RICS: Cross, Bacon, & Morris, 2000 |
| Conformity was measured with 3 items from the SCS, 2 items from the Independent Self-Construal Scale (ISS) 1 additional item. | SCS: Kagitcibasi, 2007  ISS: Singelis, 1994  Additional Item: Güngör, Karasawa, Boiger, Dinçer, & Mesquita, 2014 |
| Wellbeing | Diener, Emmons, & Griffin, 1985; Ryff & Keyes, 1995 |
| Experiences in Close Relationships | Fraley, Waller, & Brennan, 2000 |
| Emotional Support | Uchida, Kitayama, Mesquita, Reyes, & Morling, 2008 |

Table S2. Post-Laboratory Questionnaires

| **Scale** | **Source** |
| --- | --- |
| Personal Value Questionnaire | Schwartz, 2003 |
| Emotional Beliefs | Unpublished scale |
| Self Construal Scale | Hashimoto & Yamagishi, 2016 |

Table S3. Conflict topics chosen by couples

| Conflict Topic | Japan  (n = 76) | Belgium  (n = 57) |
| --- | --- | --- |
| Money or possessions relevant to your relationship (e.g. house, mortgage, car) | 18.4% | 8.8% |
| Leisure time interests or activities | 11.8% | 7.0% |
| Children (e.g. whether or not to have children, how to raise children) | 9.2% | 10.5% |
| Health (e.g. alcohol, drugs) | 13.2% | 3.5% |
| Communication (e.g. not listening to each other) | 6.6% | 12.3% |
| Relations with in-laws, family, or neighbors | 3.9% | 15.8% |
| Personal habits, characteristics, or behavioural tendencies (e.g. looks, fashion sense, diet, always showing irritation) | 10.5% | 5.3% |
| Work (e.g. time spent at work, career decisions) | 5.3% | 8.8% |
| Household-related issues (e.g. division of tasks, food) | 7.9% | 1.8% |
| Aims and goals | 5.3% | 1.8% |
| Convictions, beliefs (e.g., religion, politics), or other things believed important | 0.0% | 8.8% |
| Time spent together (e.g. amount of time, how the time is spent) | 1.3% | 5.3% |
| Major decisions (e.g. what to decide, how to decide) | 2.6% | 1.8% |
| Sex | 1.3% | 3.5% |
| Relations with friends | 1.3% | 1.8% |
| Face (e.g. losing face) | 1.3% | 0.0% |
| Role division or gender expectations | 0.0% | 1.8% |
| Showing love and affection | 0 | 1.8% |

**References**

Cross, S. E., Bacon, P. L., & Morris, M. L. (2000). The relational-interdependent self-construal and relationships. *Journal of Personality and Social Psychology*, *78*(4), 791–808. https://doi.org/10.1037/0022-3514.78.4.791

Diener, E. D., Emmons, R. A., & Griffin, S. (1985). The satisfaction with life scale. *Journal of Personality Assessment*, *49*(1).

Fraley, R. C., Waller, N. G., & Brennan, K. A. (2000). An item response theory analysis of self-report measures of adult attachment. *Journal of Personality and Social Psychology*, *78*(2), 350–365. https://doi.org/10.1037/0022-3514.78.2.350

Güngör, D., Karasawa, M., Boiger, M., Dinçer, D., & Mesquita, B. (2014). Fitting in or Sticking Together: The Prevalence and Adaptivity of Conformity, Relatedness, and Autonomy in Japan and Turkey. *Journal of Cross-Cultural Psychology*, *45*(9), 1374–1389. https://doi.org/10.1177/0022022114542977

Hashimoto, H., & Yamagishi, T. (2016). Duality of independence and interdependence: An adaptationist perspective. *Asian Journal of Social Psychology*, *19*(4), 286–297. https://doi.org/10.1111/ajsp.12145

Johnston, M. M., & Finney, S. J. (2010). Measuring basic needs satisfaction: Evaluating previous research and conducting new psychometric evaluations of the Basic Needs Satisfaction in General Scale. *Contemporary Educational Psychology*, *35*(4), 280–296. https://doi.org/10.1016/j.cedpsych.2010.04.003

Kagitcibasi, C. (2007). *Family, self, and human development: Theory and application*.

Ryff, C. D., & Keyes, C. L. M. (1995). The structure of pyschological wellbeing revisited. *Journal of Personality and Social Psychology*, *69*(4), 719–727.

Schwartz, S. H. (2003). A proposal for measuring value orientations across nations. In *Questionnaire Development Package of the European Social Survey* (pp. 259–319). https://doi.org/10.1111/j.1540-6237.2011.00830.x.Fitting

Singelis, T. M. (1994). The measurement of independent and interdependent self contruals. *Personality and Social Psychology Bulletin*, *20*(5), 580–591.

Uchida, Y., & Kitayama, S. (2001). Development and validation of a sympathy scale. *The Japanese Journal Of*, *72*(4), 275–282. https://doi.org/10.4992/jjpsy.72.275

Uchida, Y., Kitayama, S., Mesquita, B., Reyes, J. A. S., & Morling, B. (2008). Is perceived emotional support beneficial? Well-being and health in independent and interdependent cultures. *Personality and Social Psychology Bulletin*, *34*(6), 741–754. https://doi.org/10.1177/0146167208315157

Watson, D., Clark, L. A., & Tellegen, A. (1988). Development and validation of brief measures of positive and negative affect: *Journal of Personality and Social Psychology*, *54*(6), 1063–1070. https://doi.org/http://dx.doi.org/10.1037/0022-3514.54.6.1063
